# Effects of Thinning Intensity on Litterfall Production, Soil Chemical Properties, and Fine Root Distribution in *Pinus koraiensis* Plantation in Republic of Korea

**DOI:** 10.3390/plants12203614

**Published:** 2023-10-19

**Authors:** Si Ho Han, Ji Young An, Jonathan O. Hernandez, Hee Moon Yang, Eun-Sook Kim, Nam Jin Noh, Jeong Min Seo, Byung Bae Park

**Affiliations:** 1 Kasuya Research Forest, Kyushu University, Sasaguri, Fukuoka 811-2415, Japan; bupleurumhan@gmail.com; 2 Department of Environment and Forest Resources, College of Agriculture and Life Science, Chungnam National University, Daejeon 34134, Republic of Korea; johernandez2@up.edu.ph (J.O.H.); jmseo3@naver.com (J.M.S.); 3 Division of Environmental and Forest Science, College of Agriculture and Life Sciences, Gyeongsang National University, Jinju 52725, Republic of Korea; 4 Department of Forest Biological Sciences, College of Forestry and Natural Resources, University of the Philippines Los Baños, Los Baños 4031, Laguna, Philippines; 5 Division of Forest Ecology, National Institute of Forest Science, Seoul 02445, Republic of Korea; ycology@korea.kr (H.M.Y.); drummer12@korea.kr (E.-S.K.); 6 Division of Forest Science, Kangwon National University, Chuncheon 24341, Republic of Korea; njnoh@kangwon.ac.kr; 7 Division of Garden Material, Sejong National Arboretum, Korea Arboreta and Gardens Institute, Sejong 30106, Republic of Korea

**Keywords:** belowground ecology, fine root biomass, fine root necromass, Korean pine, understory vegetation

## Abstract

It is crucial to evaluate the effects of thinning on litterfall production, soil chemical properties, and fine root dynamics when implementing thinning as a silvilcultural technique to enhance tree growth and timber yield in *Pinus koraiensis* plantations. Thus, we determined the 10-year effects (2007–2017) of different thinning intensities on litterfall production, soil chemical properties, and fine root biomass and necromass within a *P. koraiensis* plantation in South Korea. The soil chemical parameters and fine root biomass and necromass were also compared across three soil depths (0–10, 10–20, and 20–30 cm). Three thinning treatments were employed: no thinning (CON), light thinning (32% removed, LT), and heavy thinning (64% removed, HT). Results revealed that litterfall was consistent across all thinning treatments, but broadleaf species had considerably higher litterfall production at HT stands than at CON/LT stands. Soil chemical properties, except exchangeable K^+^, were generally lower at LT stands, particularly at a depth of 20–30 cm soil. After ten years, there was a decrease in fine root biomass and necromass with increasing soil depth. Over 80% of fine roots were found in the upper layer (0–20 cm), while very fine roots (0–1 mm) consisted mainly of 47% pine and 53% other species and were concentrated in the 0–10 cm soil depth in HT. In conclusion, different thinning intensities had diverse effects on the parameters measured within the plantation. Future studies can explore how the effects of thinning intensities on litterfall production, soil chemistry, and fine root dynamics affect species diversity, carbon storage, and understory vegetation in *P. koraiensis* plantations.

## 1. Introduction

Thinning can minimize losses in forest carbon stocks and increase forest resiliency under warmer climates [1,2]. A changing climate not only affects plant growth but also the allocation of biomass to belowground parts, which can account for a large proportion of total carbon stored in ecosystems [3,4]. Thus, thinning has become a key forest management practice as climate change has continued to produce threats to forest growth and productivity. As a silvicultural practice, thinning can have various effects on complex belowground processes, including fine root dynamics and soil carbon and nutrient cycling in belowground ecosystems via changes in litterfall production and changes in soil physicochemical properties [5]. Fine roots are the primary organ responsible for resource acquisition and absorption in the soil, as well as resource transfer and storage, and these functions are all diameter-dependent [6,7]. Although the effects of thinning on aboveground processes are relatively well documented, its effects on belowground productivity, such as fine root production and distribution, remained unclear to date despite the important contributions to terrestrial productivity and carbon stock [8].

Litterfall is one of the important biological processes that aid carbon and nutrient cycling, energy transfer, and forest floor development [9,10]. Hence, the contribution of litterfall is critical to forest ecosystem sustainability [11]. Annual litterfall production in coniferous forests is affected by thinning intensity, with intense thinning reducing litterfall significantly, microbial C, N pools, and soil CO_2_ emissions [12]. The magnitude of the effects varies depending on thinning intensity (e.g., light and heavy thinning). Intense thinning, for example, reduced stand density and, thus, litterfall production, but accelerated nutrient release and improved nutrient availability in soil [13]. Moreover, previous studies showed that with increasing thinning intensities, understory vegetation cover and richness increased, resulting in an increase in the amount of litterfall production from different species [14]. The thinning-induced increase in the amount of litter is generally followed by changes in soil physicochemical properties and nutrient cycling [15,16]. Thinning allows more sunlight to penetrate the canopy and reach the forest floor, increasing the amount of organic materials, which has a substantial impact on soil physicochemical qualities [17]. As a short-term effect of thinning, a reduction in tree density decreases litterfall production and carbon content in the forest floor [18], and the extent may vary depending on soil depth [19]. Moreover, thinning treatments can indirectly influence belowground environmental conditions (e.g., soil fertility and microclimate), potentially impacting changes in fine-root characteristics [20,21].

Overall, thinning-induced effects on litterfall production, soil chemical properties, and fine root dynamics vary depending on ecosystem or forest type (e.g., temperate vs. Mediterranean or natural vs. plantation), thinning intensity, time elapsed from thinning, soil type, sampling heterogeneity, climatic conditions, and species composition [22,23]. Because of these several sources of variation, the pattern of results on how thinning intensity affects litterfall production, soil physicochemical properties, and fine root dynamics may be inconsistent. This uncertainty engenders the need to conduct further investigation to better explain the relationship between thinning, soil properties, and litterfall and fine root dynamics, which would provide a theoretical basis for understanding productivity and nutrient cycling in forest ecosystems, particularly plantation ones, amid climate change.

Korean pine (*Pinus koraiensis* Sieb. et Zucc.) is a significant tree species for plantation in Korea due to its high-value wood products and nuts [24]. It covers approximately 7.3% (170,905 ha) of Korea’s total forest area [25]. Ecologically, *P. koraiensis* plays an important role in forest succession, forest community structure dynamics, and biodiversity [26]. Because most forests in Korea have matured sufficiently to be adequately managed, management strategies such as thinning are implemented to enhance their growth and timber production. It is important to understand, nevertheless, that the application of thinning can have significant impacts on forest ecosystems, particularly on litterfall production, properties of soil chemistry, and fine root dynamics. In the present study, we examined the 10-year effects of different thinning intensities (control, light, and heavy) on litterfall production, soil chemical properties, and fine root biomass and necromass in a *Pinus koraiensis* plantation in South Korea. Soil chemical parameters, as well as fine root biomass and necromass, were compared across three soil depths (0–10, 10–20, and 20–30 cm). We hypothesized that: (1) increasing thinning intensity would result in a decrease in litterfall production; (2) increasing thinning intensity and decreasing soil depth would result in an increase in fine root biomass and necromass; and (3) changes in soil chemical parameters would be more pronounced with heavy thinning and shallower soil depth.

## 2. Results

### 2.1. Litterfall Production by Thinning Intensity

**Figure 1:**
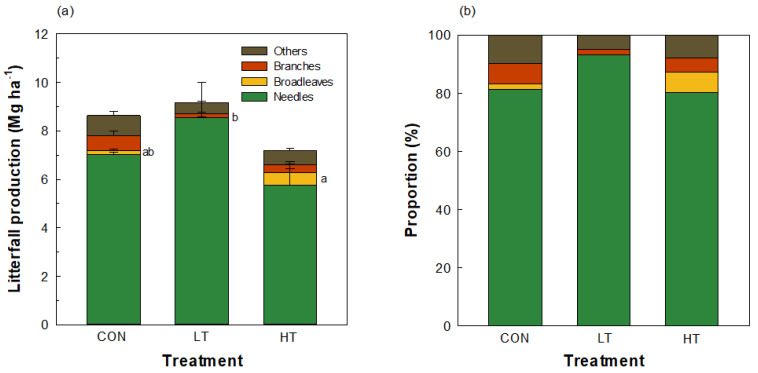
(**a**) Annual litterfall production and (**b**) proportions of each component in the *Pinus koraiensis* plantation 10 years after thinning (CON, control (no thinning); LT, light thinning (32% removed); HT, heavy thinning (64% removed)). Needles indicate leaf litterfall of *P. koraiensis*. Broadleaves include leaf litterfall of other species except *P. koraiensis*. Branches include twigs. Others indicate all tissues except leaves and branches. Vertical bars represent the standard error (*n* = 4). Means of broadleaves with different lowercase letters indicate significant differences between treatments (thinning intensities) in one-way analysis of variance (ANOVA) based on Tukey’s studentized range (HSD) test. Means without lowercase letters were not significantly different.

In this study, the total production of litterfall ranged from 7.19 to 9.17 Mg ha^−1^, and no significant differences were found by thinning intensities (*p* > 0.05) (Table S1). The needle litterfall of *P. koraiensis* constituted 85% of the total litterfall production on average. Branch and other tissues exhibited the highest production in the CON. Although the annual litterfall production of needles, branches, or other tissues did not display significant variation between treatments, litterfall production of broadleaf species at the HT was significantly greater by 150% (*p* = 0.027) compared to the LT (Figure 1).

### 2.2. Thinning-Induced Changes in Soil Chemical Properties

In the present study, there was no significant interaction between treatment and soil depth (*p* > 0.05). However, there were significant variations in soil chemical properties across thinning intensities and soil depths after ten years of thinning (Table 1). Overall, soils within *P. koraiensis* plantations were acidic (4.84 to 5.25) regardless of thinning intensities. Total nitrogen (TN) tended to be higher at the HT by 72% and 148% compared to the CON and LT treatments, respectively. At a soil depth of 10–20 cm, organic matter (OM) was 81% higher at the HT than LT. The LT stand exhibited significantly lower levels of most exchangeable cations (excluding K^+^) compared to the other two stands. This trend was also observed for available phosphorus (AP) and cation exchange capacity (CEC), particularly at 0–10 and 10–20 cm soil depth.

### 2.3. Vertical Distribution of Fine Roots in Different Thinning Intensity Treatments

**Figure 2:**
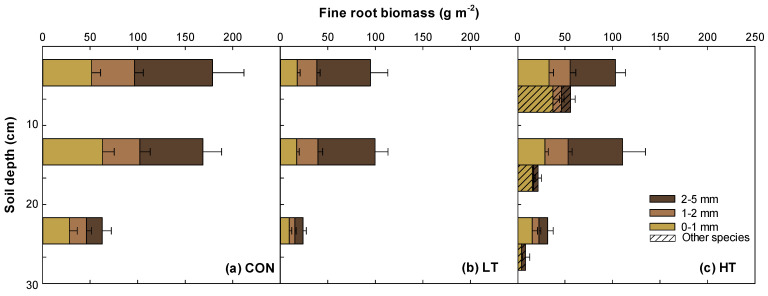
Vertical distribution of fine root biomass in the *Pinus koraiensis* plantation 10 years after thinning ((**a**) CON, control (no thinning); (**b**) LT, light thinning (32% removed); (**c**) HT, heavy thinning (64% removed)). Other species indicates fine roots of understory vegetation except *P. koraiensis*. Fine roots of other species were not observed in the control and light thinning stand. Vertical bars show standard errors (*n* = 8).

Fine root biomass varied significantly across thinning intensities for both pine (*p* = 0.0002) and other species (*p* < 0.0001) (Figure 2, Table S2). The overall root biomass (0–5 mm) was the highest in the CON treatment, followed by HT and LT. In the soil depth of 0–30 cm, total root biomass (0–5 mm) was the highest at CON (410.16 g m^−2^), intermediate at HT (245.75 g m^−2^), and the lowest at LT (218.38 g m^−2^) for pine species. Interestingly, roots of other species were only found at the HT treatment, with a value of 86.55 g m^−2^. The very fine roots (0–1 mm) of other species were approximately double the quantity of bigger fine roots (1–5 mm), mainly distributed in the upper soil layer. Meanwhile, roots from other species were not seen in both CON and LT treatments.

**Figure 3:**
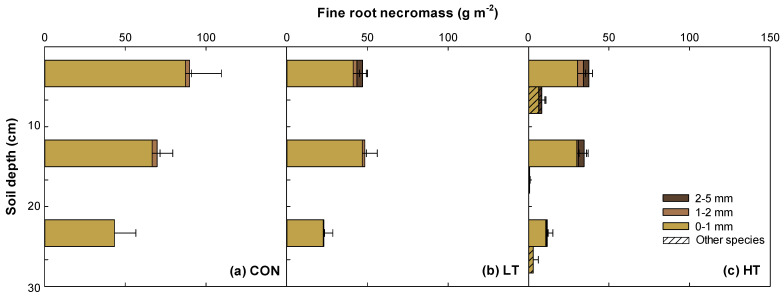
Vertical distribution of fine root necromass in the *Pinus koraiensis* plantation 10 years after thinning ((**a**) CON, control (no thinning); (**b**) LT, light thinning (32% removed); (**c**) HT, heavy thinning (64% removed)). Other species indicates fine roots of understory vegetation except *P. koraiensis*. Fine roots of other species were not observed in the control and light thinning stand. Vertical bars show standard errors (*n* = 8).

A similar pattern was observed for fine root necromass (Figure 3, Table S2). Total root necromass (0–5 mm) was highest in the CON treatment (202.84 g m^−2^), followed by LT (118.49 g m^−2^) and HT (96.78 g m^−2^) treatments. The fine root necromass of other species was solely detected in the HT stand, representing approximately 13% of the total root necromass.

With regard to vertical root distribution pattern ten years post-thinning, there was a decrease in fine root biomass as soil depth increased by 43–65% at CON, 47–82% at LT, and 44–69% at HT (Figure 2). Similarly, as soil depth increased, there was a general reduction in fine root necromass of pines by 41–52% at CON, 32–72% at LT, and 37–74% at HT (Figure 3). A comparable trend can be seen in the fine root biomass and necromass of other species in the HT stand. The majority (>80%) of these fine roots were consistently distributed in the upper soil layer (i.e., 0–20 cm). For very fine roots (0–1 mm), approximately 47% pine and 53% other species were distributed primarily within the 0–10 cm soil depth.

## 3. Discussion

### 3.1. Significant Increase in Broadleaf Litterfall Production in a Pinus koraiensis Plantation Ten Years after Thinning

Thinning is an important forest management practice with the potential to influence the overall forest dynamics, as reflected by significant changes in litterfall production between needle and broadleaf species ten years after thinning in the present study. Similarly, a previous study found that selective thinning significantly reduced conifer fractions of litterfall (c.a., 48%), whereas litterfall of deciduous species increased by at most 20% [11]. Another report also estimated that intense thinning reduced needle litterfall fractions [12]. Higher litterfall production of broadleaf species at heavily thinned *Pinus koraiensis* plantation than the lightly thinned stand is likely due to a combination of altered environmental conditions, including signification alterations in light conditions [27] and soil conditions [28]. In a thinned Sitka spruce stand, the light transmittance increased exponentially as the basal area was reduced significantly [27]. Increased light availability can result in the increased growth and survival of understory vegetation, thereby increasing the diversity of plant species [29,30,31] and enhancing nutrient cycling and ecological habitat factors [32]. This strongly supports the previous reports that thinning practices can, directly and indirectly, affect the formation of understory vegetation [33,34]. A similar pattern was reported in Yu et al. [32], wherein there was a significant increase in shrub diversity following thinning.

Because of thinning-induced environmental changes, thinning treatments can promote the natural regeneration of broadleaf species, resulting in an increase in their presence and litterfall over time. In the present study, the proportion of needle litter at HT stand tended to decrease with increasing broadleaf litter because of species-specific differences [35]. The result suggests that pine and broadleaf species may have different responses to thinning, and the latter may be more responsive to the altered environmental conditions. This could be due to their difference in life history traits (e.g., growth rates, shade intolerance, functional type). A previous study found a gradual increase in hardwoods, which are generally produced by broadleaf species within a red pine plantation due to differences in dispersal traits, seed characteristics, and relative adaptation to site conditions [36]. Moreover, regeneration of oak species tends to be challenging under broadleaf canopies but often demonstrates success when situated under pine canopies [37,38]. This phenomenon will likely increase the proportion of litterfall from broadleaf species within the *P. koraiensis* plantation over time.

Generally, broadleaf species also have better regenerative traits compared with many pine species because they can generally regenerate in various ways, such as stumps, root systems, and acorns, which are effective for seed dispersal [39,40,41]. Despite limited light availability due to the dense pine canopy at CON, broadleaf species were able to regenerate successfully, resulting in an increased litter input. The light environment at lightly thinned stands, on the other hand, may not have benefited broadleaf species regeneration because the environment may have supported the competitive advantage of pines instead, suppressing broadleaf species establishment. This supports the previous study’s findings that a higher height growth of pine plantations was recorded in very low and low thinning intensity treatments than in heavy thinning treatment [42]. This validates the current study’s findings of lower soil chemical properties (N, AP, CEC, Ca^+^, and Mg^+^) at LT than HT and CON at the 10–20 cm soil depth. The reduced presence of broadleaf species or a higher proportion of needle litter from *P. koraiensis*, which has litter that decomposes slower than broadleaf species [43], can be attributed to the slower nutrient cycling at LT stand. A study found that needle-leaf litter had lower N, P, K, and Mg contents than broadleaf litter [44]. The authors further noted that the decomposition rates of these litters were positively correlated with their initial elemental contents. Consequently, the greater proportion of broadleaf litter at CON and HT compared to LT provides another explanation for the much higher soil chemical characteristics seen at CON and HT.

### 3.2. Heavy Thinning Intensity Increased Abundance of Other Species’ Roots in P. koraiensis Plantation

Pine trees are known to be dominating competitors for important resources such as light, water, and nutrients [45], and, thus, their removal may have reduced intraspecific competition within the HT stand [46]. This provided other species with greater possibilities to establish and expand their root systems. This helps explain why there was a higher proportion of litter from broadleaf species at the HT stand. Reduced competition for light, for instance, benefits understory plants that can now photosynthesize more efficiently and allocate more resources to their root systems. This helps explain why there was a higher proportion of pine roots at CON than any thinned stands in the present study. A dense pine canopy could have significantly decreased major competitors of *P. koraiensis,* thereby resulting in the development of well-established root systems of the species. This shows that even in the presence of competing vegetation (most likely shade-tolerant) at the CON stand *P. koraiensis* can develop extensive roots, enabling effective access to water and nutrients in the soil, provided the stand has not been disturbed. Moreover, results imply that the CON stand may have had a more competitive environment, favoring the abundance of pine roots over other species in the plantation. Hence, the removal of the dense canopy of *P. koraiensis* at the HT stand may have provided other species (most likely shade-intolerant) with a higher light supply reaching the forest floor, enabling their growth and the establishment of their roots. A similar study found that the <0.5 mm fine roots fluctuated with increasing thinning intensities [8].

### 3.3. Vertical Distribution Patterns of Fine Root Biomass and Necromass in P. koraiensis Plantation

The pattern in the vertical distribution of fine root biomass and necromass observed in *P. koraiensis* plantation can be ascribed to soil conditions, particularly water and nutrient contents in different soil layers [47,48], as reported in previous studies [49,50,51]. Specifically, as soil depth increases, soil water and nutrient resources become more limited, which can lead to a decrease in fine root production and, consequently, a decrease in fine root necromass buildup in a particular soil depth. This phenomenon is evident in the distribution of very fine roots (0–1 mm) of other species, with the majority localized in a shallower soil depth of 0–10 cm, particularly at HT stand. Because organic matter, nutrients, and moisture are often more concentrated in the top 10 cm of soil, very fine roots tend to establish in that depth range to effectively access essential resources. Very fine roots are more dynamic and sensitive to soil water and nutrient changes than thicker roots [8,52]. Conversely, a high abundance of very fine roots in a shallower soil depth of 0–10 cm may have contributed organic matter to soil by shedding roots and exudates, which can encourage microbial activity, enhancing organic matter decomposition and mineralization. This further supports the observed higher OM and TN contents at 0–10 cm soil depths compared with 10–20 and 20–30 cm soil depths in all treatments. Moreover, increased surface litter buildup over time can lead to greater OM and/or TN levels at the 0–10 cm soil depth, regardless of thinning intensity. A study also found that soil nutrient status (e.g., N) was related to fine root necromass in pine plantations [53]. The fine root distribution pattern is controlled by soil resource availability, with a positive correlation reported with soil organic carbon [54]. Localized distribution of very fine roots of other species in a shallower soil depth of 0–10 cm may have also caused localized changes in soil pH because plant roots generally release organic acids (H^+^) that make the soil more acidic, counteracting the effect of charge imbalance as a result of increased nutrient uptake by fine roots [55].

### 3.4. Ecological Implications

The findings of the present study provide implications for several aspects of forest ecology and management, such as biodiversity, root species composition, soil health, and nutrient cycling, all of which play important roles in shaping plantation productivity [56,57]. The increased litterfall production of broadleaf species in HT stands indicates the ecological significance of biodiversity in forest plantation ecosystems. Thus, the application of the HT method is especially useful when the management purpose is to address the limitations of mono-species plantations. An experiment revealed complementarity in different species’ litterfall dynamics that resulted in consistent litter supply in species-rich stands [58]. This means that by opting for heavy thinning, the environmental conditions that can potentially favor the growth and dominance of broadleaf species can be modified, at least in the case of *P. koraiensis* plantation. The use of HT treatment can also be useful when the management purpose is to improve the forest’s overall health and productivity, as a broad mix of tree species can contribute to healthier and more resilient plantations. It is important to note that only reasonable forest thinning management can result in an increased proportion of broadleaf litter via an increase in species diversity, tree growth, and productivity [59].

The application of HT method within the *P. koraiensis* plantation can contribute to a more dynamic nutrient cycling mechanism through an increased diversity in litter quality, contributing different organic matter and nutrients to the soil for plant growth [32,60]. This is because broadleaf litter generally decomposes faster than those of the other litter types (e.g., coniferous litter). Such a faster decomposition can accelerate the release of macronutrients, particularly nitrogen, phosphorus, and potassium, into the soil as they become easily available for plant absorption. This can result in a more stable soil structure as the litter decomposes over time. It can also help mitigate soil acidification since coniferous litter generally accumulates acidic compounds over time due to a slower decomposition rate. The presence of a high amount of broadleaf litter can neutralize soil acidity by introducing a wider range of organic matter in the soil [61]. Hence, our findings are relevant to managing pine forest plantations encountering acidity issues, which influence different soil biological and physicochemical processes that affect timber yield.

Moreover, the observed root pattern in HT stands, with very fine roots primarily made of roots from other species, suggests that various tree species may have different root strategies in response to the altered environmental conditions (e.g., resource availability and light conditions) caused by thinning treatments. These strategies can contribute to resource partitioning between pine and broadleaf species in the plantation, influencing competition for resources. This is because, depending on the environmental conditions, some tree species may allocate more resources to fine roots while others may choose deeper root systems [62,63].

## 4. Materials and Methods

### 4.1. Site Descriptions

**Figure 4:**
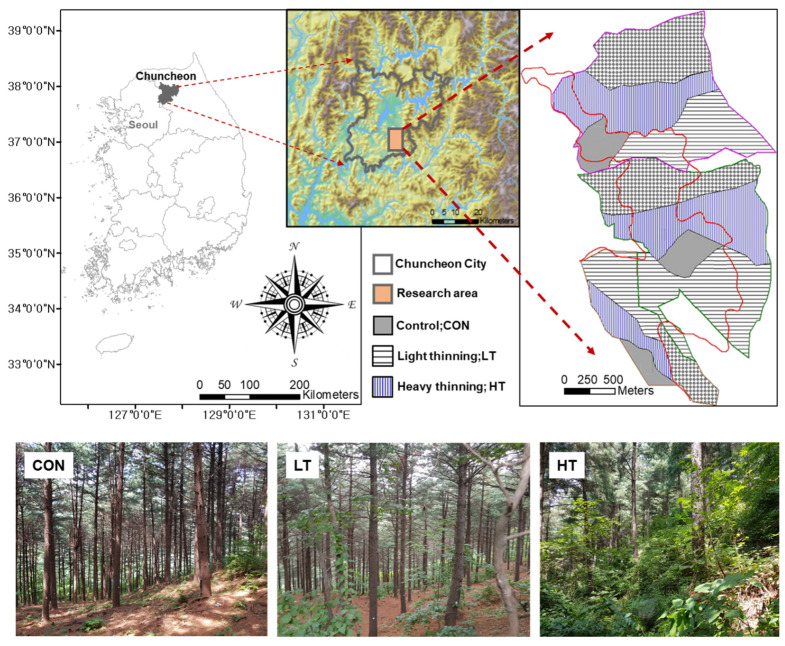
Locations of CON (control or no thinning), LT (light thinning) and HT (heavy thinning) stands in *Pinus koraiensis* plantation at Mt. Gari, Chuncheon-si, Gangwon-do, South Korea.

This study was conducted in the *Pinus koraiensis* plantation at Mt. Gari, Chuncheon-si, Gangwon-do of South Korea (37°53′ N, 127°52′ E) with a mean elevation of approximately 367 m above sea level and a mean slope of 25° (Figure 4). The area of the plantation was 117 ha, where *P. koraiensis* seedlings (Korean pine) were planted in the 1960s (c.a., 60 years old already in 2023).

**Figure 5:**
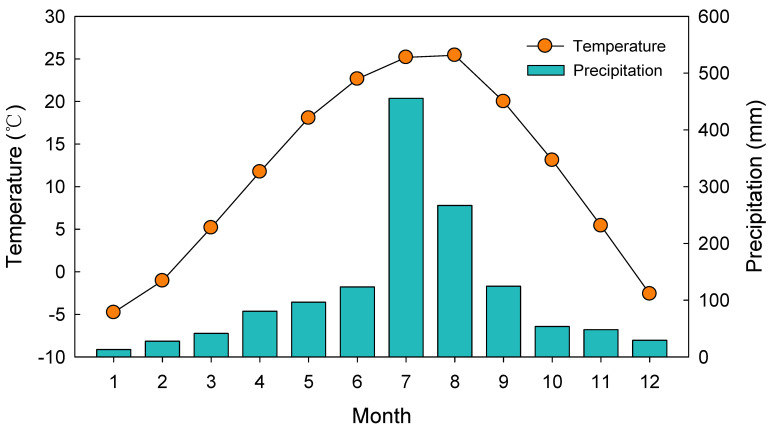
The study site’s annual mean temperature and precipitation from 2007 to 2018.

The soil in the study site is classed as dry brown forest soil (B1) with shallow depth and low organic content. The mean annual temperature was 11.5 °C and the average annual precipitation was 1360 mm from 2007 to 2018 (Figure 5).

Here, four plots measuring 20 × 20 m per treatment were established in the study site. Three thinning treatments were employed in 2007, namely, no thinning (CON), light thinning (LT, 32% removed), and heavy thinning (HT, 64% removed) (Table 2). These thinning treatments were determined based on stand characteristics, such as diameter at breast height (DBH), height, and basal area, of *P. koraiensis* plantation before and after thinning operations (Table 2).

### 4.2. Estimation of Annual Litterfall Production

Two circular litter traps (0.25 m^2^) were set 1 m above ground at 10 m intervals within a 20 × 20 m plot (*n* = 4) to analyze litterfall production across different thinning treatments. Litterfall production was collected from June 2016 to May 2017 (10 years after the first survey) at a 3-month interval. The samples were air-dried under shade and then classified into different plant components. The litterfall sample was classified as *P. koraiensis* needle leaf (needles), understory litter fall (broadleaves), branches, and others. Sorted samples were oven-dried at 65 °C for 72 h and then measured for dry weight.

### 4.3. Soil Sampling and Analyses

Soil sample collection was carried out from randomly selected undisturbed sites, comprising four plots per treatment, for soil chemical property determination. For each selected site, a 0.5 kg sample was collected from each soil depth (i.e., 0–10, 10–20, 20–30 cm). Visible roots and other objects from the sample were first removed before homogenizing them into a composite sample by plot. Soil acidity (pH) was measured by diluting 10 g of soil in distilled water at a ratio of 1:5. Available phosphate was determined by the Lancaster method, and total nitrogen was determined by the micro-Kjeldahl method using 1 g of soil sample. The exchangeable cations K^+^, Ca^2+^, and Mg^2+^ were extracted using 1 N’s NH_4_OAc and measured using an Atomic Absorption Spectrometer (AA280FS, Agilent Technologies, Wood Dale, IL, USA). Cation exchange capacity (CEC) was determined by the Brown method after extraction with 1 N’s of HN_4_OAc and CH_3_COOH solution.

### 4.4. Measurement of Vertical Distribution of Fine Roots

In this study, stainless steel soil cores (length: 60 cm, diameter: 5 cm) were used to obtain the soil fine roots of *P. koraiensis*. Two points were selected at a 10-m interval within the 20 × 20 m plot, and two soil cores were collected from each point at a 2-m interval. Samples were obtained at a depth of 30 cm during summer in August. A soil probe was first utilized for sampling to avoid large roots, and rocks, among others in the soil. Root core samples were collected from mineral soil, excluding the organic layer, in three soil depths (0–10, 10–20, 20–30 cm). Samples from each site were mixed by depth within the survey area (eight replicates per treatment) and stored at the laboratory at low temperature for further analysis. Thereafter, all fine roots of different diameters were carefully sorted. Collected fine roots were washed under running water, and dead fine roots were identified based on fine root color, recoverability, and firmness of the rhizodermis and xylem [15]. Samples were divided into live and dead fine roots, and the live fine roots were further categorized into three diameter classes (0–1, 1–2, 2–5 mm). Dead fine roots were classified into two diameter classes (0–2, 2–5 mm). Samples were further divided into either pine tree roots or fine roots of other understory vegetation. Sorted fine roots were dried in a desiccator at 65 °C up to constant weight before measuring the dry weight.

### 4.5. Statistical Analyses

We conducted a one-way ANOVA to test for significant differences among thinning treatments for each type of litterfall tissue. A two-way ANOVA was used to assess the effect of treatments and soil depths on soil chemical properties. By checking for interactions and finding none, one-way ANOVA was performed for each soil chemical property at the three soil depths to identify which thinning intensities significantly affect each property at specific soil depths. For fine roots, the interaction of treatment, soil depth, and root diameter on fine root distribution was investigated using a three-way ANOVA to determine how these factors interact and whether they collectively influence fine root distribution. Also, a one-way ANOVA for each species was conducted separately to test for significant differences among treatments in terms of fine root biomass and necromass. Thereafter, Tukey’s HSD test was used as a post-hoc analysis. All statistical tests were performed using the SAS 9.4 program at a 5% significance level.

## 5. Conclusions

In conclusion, the 10-year study on different thinning intensities in a *Pinus koraiensis* plantation in South Korea found that increasing thinning intensity resulted in a significant increase in litterfall production of broadleaf species. Soil chemical properties generally showed lower values at LT stands except for exchangeable K^+^. A decade following thinning, a notable change in fine root composition due to the heavy thinning treatment was observed. The majority of fine roots (>80%) are localized in the upper 0–20 cm soil layer, and roughly 47% of very fine roots belong to pine, while the remaining 53% are from other species, mostly found within the 0–10 cm soil level. When using thinning as a silvicultural method to increase tree growth and timber production in *P. koraiensis* plantations, it is critical to examine the impact of thinning on litterfall generation, soil chemical characteristics, and fine root dynamics.

## Figures and Tables

**Table 1:** Soil chemical properties at different soil depths (0–10, 10–20, 20–30 cm) and treatments (control, light thinning, heavy thinning) in the *Pinus koraiensis* plantation of South Korea, 10 years after thinning.

Soil Depth	Treatment	pH	EC	OM	TN	AP	CEC	Exchangeable Cations (cmolc kg^−1^)		
(cm)			(dS m^−1^)	(%)	(%)	(mg kg^−1^)	(cmolc kg^−1^)	K^+^	Ca^2+^	Mg^2+^
0–10	CON	4.95	0.49	5.93	0.29	29.30	10.83	0.19	3.14	2.05
		(0.06)	(0.02)	(1.01)	(0.02)	(5.73)	(0.63)	(0.005)	(0.51)	(0.09)
	LT	4.97	0.28	4.09	0.26	9.86	8.19	0.19	1.27	1.62
		(0.08)	(0.03)	(0.28)	(0.01)	(3.50)	(0.27)	(0.003)	(0.42)	(0.12)
	HT	5.25	0.58	5.44	0.34	22.92	10.82	0.19	3.95	2.27
		(0.05)	(0.08)	(0.41)	(0.01)	(3.25)	(0.63)	(0.006)	(0.47)	(0.10)
10–20	CON	4.88	0.34	2.77	0.13	8.98	8.83	0.19	1.71	1.77
		(0.11)	(0.03)	(0.56)	(0.06)	(1.67)	(0.47)	(0.003)	(0.31)	(0.07)
	LT	4.85	0.22	1.91	0.05	4.85	7.05	0.18	0.51	1.41
		(0.08)	(0.02)	(0.09)	(0.00)	(1.71)	(0.24)	(0.00)	(0.25)	(0.06)
	HT	5.23	0.40	3.45	0.28	12.27	8.79	0.19	2.47	1.96
		(0.04)	(0.04)	(0.07)	(0.00)	(1.63)	(0.40)	(0.003)	(0.08)	(0.12)
20–30	CON	4.84	0.30	1.97	0.08	12.70	8.06	0.18	1.24	1.64
		(0.08)	(0.03)	(0.34)	(0.04)	(6.47)	(0.16)	(0.00)	(0.23)	(0.07)
	LT	4.84	0.19	1.47	0.04	4.61	7.38	0.18	0.62	1.47
		(0.08)	(0.01)	(0.16)	(0.01)	(0.84)	(0.38)	(0.00)	(0.35)	(0.08)
	HT	5.16	0.31	2.30	0.24	7.57	8.30	0.18	2.22	1.89
		(0.02)	(0.03)	(0.22)	(0.00)	(1.52)	(0.62)	(0.00)	(0.19)	(0.13)
	*p*-values									
	Treatment	**<0.0001**	**<0.0001**	**0.004**	**<0.0001**	**0.003**	**<0.0001**	0.33	**<0.0001**	**<0.0001**
	Soil depth	0.19	**<0.0001**	**<0.0001**	**<0.0001**	**0.0002**	**<0.0001**	**0.001**	**<0.0001**	**0.001**
	T × D	0.98	0.23	0.53	0.08	0.20	0.27	0.88	0.40	0.66

Values in parenthesis represent the mean and standard errors (*n* = 4). *p* values represent the result of two-way analysis of variance (ANOVA) for examining the effects of treatments and soil depths on soil chemical properties. T × D indicates the interaction between treatment and soil depth. Significant *p*-values are written in bold font. Different lowercase letters indicate significant (*p* < 0.05) differences among treatments (thinning intensities) at three soil depths in one-way analysis of variance (ANOVA) based on Tukey’s studentized range (HSD) test. CON, control (no thinning); LT, light thinning (32% removed); HT, heavy thinning (64% removed). OM, organic matter; TN, total nitrogen; AP, available phosphorus; CEC, cation exchange capacity.

**Table 2:** Stand characteristics of *Pinus koraiensis* plantations subjected to different thinning intensities, namely, no thinning (CON), light thinning (LT, 32% removed), and heavy thinning (HT, 64% removed) in 2007 at Mt. Gari, Chuncheon-si, Gangwon-do (37°53′ N, 127°52′ E). Tree inventory was done in 2007 (before thinning) and in 2017 (10 years after thinning).

		Before Thinning						10 Years after Thinning					
		CON		LT		HT		CON		LT		HT	
Stem density	(trees ha^−1^)	538		588		550		500		363		188	
DBH	(cm)	24.2	(0.5)	22.3	(0.6)	21.9	(0.4)	31.5	(0.7)	32.5	(0.7)	36.0	(0.8)
Height	(m)	15.1	(0.3)	13.4	(0.2)	14.5	(0.2)	19.4	(0.2)	18.3	(0.2)	17.4	(0.2)
Basal area	(m^2^ ha^−1^)	24.7		23.0		20.7		39.7		30.5		19.2	

## Data Availability

All the data used are already reflected in the article. Other relevant data may be available upon request from the authors.

## Supplementary Materials

The following supporting information can be downloaded at: https://www.mdpi.com/article/10.3390/plants12203614/s1, Table S1. Summary of one-way and two-way ANOVA results for the effects of thinning treatments on litterfall; Table S2. Summary of the two-way and three-way ANOVA results for the effects of thinning treatments, soil depth, and their interactions on fine root biomass and necromass.
