# Supplementary material for: Effects of Thinning Intensity on Litterfall Production, Soil Chemical Properties, and Fine Root Distribution in Pinus koraiensis Plantation in Republic of Korea
Source: Plants (Basel). 2023 Oct 19;12(20):3614. doi: 10.3390/plants12203614 (PMC10609674; doi:10.3390/plants12203614)
Supplement: Supplementary file 1 [file plants-12-03614-s001.zip › plants-2676410-supplementary.pdf]

Supplementary Material

Table S1. Summary of one-way and two-way ANOVA results for the effects of thinning treatments on litterfall.

Treatment	DF	P values
Needles	2	0.149
Broadleaves	2	0.028
Branches	2	0.186
Others	2	0.097
Total	2	0.353

Table S2. Summary of the two-way and three-way ANOVA results for the effects of thinning treatments, soil depth, and their interactions on fine root biomass and necromass.

Treatment	DF	P	Treatment	DF	P values
**Fine root biomass**			**Fine root necromass**		
**Pine species**					
**Three-way ANOVA**					
Diameter	2	<0.001	Diameter	2	<0.001
Treatment x Diameter	4	0.634	Treatment x Diameter	4	<0.001
Depth x Diameter	4	0.036	Depth x Diameter	4	0.003
Treatment x Depth x Diameter	8	0.975	Treatment x Depth x Diameter	8	0.765
**Two-way ANOVA**					
Treatment	2	<0.001	Treatment	2	<0.001
Depth	2	<0.001	Depth	2	0.002
Treatment x Depth	4	0.781	Treatment x Depth	4	0.831
**Other species**					
**Three-way ANOVA**					
Diameter	2	<0.001	Diameter	2	0.065
Treatment x Diameter	4	<0.001	Treatment x Diameter	4	0.028
Depth x Diameter	4	0.003	Depth x Diameter	4	0.693
Treatment x Depth x Diameter	8	<0.001	Treatment x Depth x Diameter	8	0.811
**Two-way ANOVA**					
Treatment	2	<0.001	Treatment	2	0.012
Depth	2	<0.001	Depth	2	0.270
Treatment x Depth	4	<0.001	Treatment x Depth	4	0.264
**Total**					
**Three-way ANOVA**					
Diameter	2	<0.001	Diameter	2	<0.001
Treatment x Diameter	4	0.237	Treatment x Diameter	4	<0.001
Depth x Diameter	4	0.081	Depth x Diameter	4	0.002
Treatment x Depth x Diameter	8	0.779	Treatment x Depth x Diameter	8	0.787
**Two-way ANOVA**					
Treatment	2	0.001	Treatment	2	<0.001
Depth	2	<0.001	Depth	2	0.001
Treatment x Depth	4	0.730	Treatment x Depth	4	0.855
